# Strength and Speed Profiles Based on Age and Sex Differences in Young Basketball Players

**DOI:** 10.3390/ijerph18020643

**Published:** 2021-01-13

**Authors:** David Mancha-Triguero, Javier García-Rubio, José M. Gamonales, Sergio J. Ibáñez

**Affiliations:** GOERD Group, Faculty of Sport Sciences, University of Extremadura, 10003 Cáceres, Spain; jagaru@unex.es (J.G.-R.); martingamonales@unex.es (J.M.G.); sibanez@unex.es (S.J.I.)

**Keywords:** physical fitness, performance profile, jumps, repeat sprint ability tests (RSA), inertial devices, sex differences, age differences

## Abstract

Team sports are in continuous evolution, and physical performance is acquiring greater importance in the game. The assessment of physical fitness is the most reliable method for knowing if the athlete’s physical fitness is appropriate. Therefore, the objectives of this research were to identify profiles of physical-physiological demands with different specific tests of physical fitness related to the maximum strength of the lower body and speed. Moreover, some differences based on the sex and age of the players were identified. One hundred and forty-nine basketball players were analyzed (men *n =* 103 vs. women *n =* 46, weight: 74.74 ± 11.65 kg vs. 56.89 ± 3.71 kg, height: 184.66 ± 11.67 vs. 164 ± 4 and BMI: 21.7 ± 0.83 vs. 21.6 ± 0.90). The players performed an Abalakov test, a multi-jump test, and a repeat sprint ability test. Each player was equipped with a WIMU PRO device, and photoelectric cells were used. A MANOVA was performed to discover the differences between ages, and a *t*-test was used regarding sex. The results showed significant differences based on age and sex in variables related to time and Player Load/min (*p* < 0.001). The variables related to steps (contact, step, flight) also showed significant differences as a function of age (*p* < 0.001) and sex (*p* < 0.05). The multi-jump test showed differences based on age (*p* < 0.05 except in between jumps), but not on sex. These results confirm the importance of working together on lower body strength and speed skills. In addition, the planning of the work on these abilities must be individualized and according to the characteristics of the athlete.

## 1. Introduction

Basketball is an intermittent and multifaceted team sport [1] that demands a wide range of physical attributes, including the ability to perform repeated sprints, jumps, and high-intensity runs [2]. As in the rest of team sports, the improvement of the player’s performance must be approached in a comprehensive manner (including the physical fitness of the athlete) [3]. The physical fitness of the player will be influenced by the type of practice to be carried out and should be optimized according to the specificity of the sport. Basketball is an aerobic-based sport [4] composed of different high-intensity anaerobic actions [5]. This characteristic has undergone an evolution in terms of the demands that the player supports and the intensity of the competition. In this respect, basketball is a continuously evolving sport, implying an increase in the intensity, number and duration of explosive actions [6]. This evolution means that the physical condition of the athlete also evolves to meet the requirements demanded by the sport, therefore improving the level of competition and entertainment.

Aerobic capacity is essential for the players to face the duration of the match. In addition, it helps them to recover more quickly and efficiently from anaerobic efforts [7]. The player’s anaerobic performance is the most important descriptor of the final result [8]. Anaerobic performance is classified into anaerobic capacity and power [9]. The anaerobic capacity allows the athlete to derive energy from a combination of anaerobic glycolysis and the phosphagen system, while anaerobic power reflects the ability to use the phosphagen system [9], understood as the relationship between the application of force and speed in an action of maximum intensity for a short time [10]. Within this last factor, strength is the fundamental pillar on which this sport is based [11]. In this case, not only strength but related abilities (speed, agility, change of direction) play an important role in the game [12] because the nature of the sport is intermittent, unpredictable and chaotic [1]. Along these lines, the application of force in basketball players has been studied mainly in the lower part of the body [13].

Traditionally, generic laboratory tests related to the 1RM (test of 1 repetition maximum) have been used to assess maximum lower body strength in athletes. Currently, there is research that relates generic laboratory strength tests with field or sport-specific tests. To the best of our knowledge, there is hardly any research that only makes evaluations of the strength of basketball players through specific field tests, such as jumping, sprinting or repeat sprint ability tests (RSA) [14,15]. This type of test is characterized by performing a set of repeated sprints with a personalized recovery period depending on the objective. All these actions improve and optimize the athlete’s performance and have an impact on the competition itself due to its specificity [16].

Regarding sex-related differences, previous research has confirmed that the number of type II muscle fibers is higher in men [17,18]. In addition, there are differences in muscle mass, strength, and muscle quality [19]. These individual characteristics influence explosive actions or gestures since they are responsible for generating greater contractile force and speed [20]. The differences are also found in the function of age (age-related) since it is a quality that evolves as a function of the evolutionary development of the athletes [21]. Regarding research found in young basketball players, differences in the strength generated by the lower body were observed in a cross-sectional study as a function of age and sex [22]. In addition, in 11–13-year-olds, notable differences were not found. These differences appeared in the 15–17 years age group [22] in the force generated in the lower body, confirming that women players presented lower values in relation to body weight (relative strength). Regarding the differences related to age, [21] it was confirmed that, in young basketball players, the period of puberty is limiting in the development of skills, since the appearance of this physiological process modifies the development of the physical abilities of women’s basketball players and therefore their performance, with considerable differences with respect to men.

In the sports field, it is common to present information through profiles. This way of presenting the results is because it facilitates the graphic representation and the understanding of variables that provide a comprehensive vision of the object of study (evaluated through different variables) and that have different scales [23]. In addition, it is a common practice both in the field of research and in the professional field of training for the study of performance indicators [24,25,26,27,28] or the evaluation of physical condition [29].

A review of the literature reveals the existence of a relationship between the maximum force of the lower body and the sprint (transfer of maximum force). However, it is necessary to corroborate that the scientific evidence revealed in professional players on the knowledge of how the maximum strength of the lower body is identified and the transfer of this force in speed also coincides with that of basketball players in training categories (U14, U16 and U18). Furthermore, there are few studies that use data from the latest generation of microsensor instruments in basketball players, and fewer still in young players [29,30]. Therefore, the objectives of this research were to identify profiles of physical-physiological demands with different specific tests of physical fitness related to maximum strength and speed of the lower body. In addition, it was aimed to compare the differences based on the sex and age of the players. Furthermore, the hypothesis of this research was that men players would present better results than women players in all the tests carried out and that older players, when faced with the same stimulus (test), would obtain better results than younger players in all variables.

## 2. Materials and Methods

### 2.1. Design

This study was carried out following a cross-sectional design that involved testing each participant once.

### 2.2. Participants

One hundred and forty-nine players, both men and women of different ages (U14, U16 and U18), who participate in the national championship, were analyzed (U14 men: *n =* 33, weight *=* 62.20 kg, height *=* 1.72 m, BMI *=* 20.78; U14 women: *n =* 12, weight *=* 53 kg, height *=* 1.60 m, BMI *=* 21.875; U16 men: *n =* 31, weight *=* 76.81 kg, height *=* 1.87 m, BMI *=* 21.91; U16 women: *n =* 12, weight *=* 60.39 kg, height *=* 1.64 m, BMI *=* 22.34; U18 men: *n =* 39, weight *=* 85.23 kg, height *=* 1.95 m, BMI *=* 22.41; U18 women: *n =* 22, weight *=* 57.3 kg, height *=* 1.68 m, BMI *=* 20.59). The best teams of the regional championships informative categories play in the National Championship of Spain. The number of players by age and sex corresponds to the components of various teams. The number of members of each basketball team ranges from 10 to 12 players. The reality of sports training implies that the number of players analyzed cannot provide very large samples [31]. To be part of the selected sample, the players participated in the whole design proposed and were not able to be part of the sample if they did not complete any one of the tests in the study. The coaches, players and parents of underage players were previously informed of the details of the investigation and of its possible risks and benefits; the participation of the athletes was voluntary. For this, approval of participation was requested through informed consent. In underage players, the consent was signed by their legal guardians. The study was developed based on the ethical provisions of the Declaration of Helsinki (2013) and was approved by the Bioethics Committee of the University (n° 233/2019).

### 2.3. Variables

**Figure 1:**
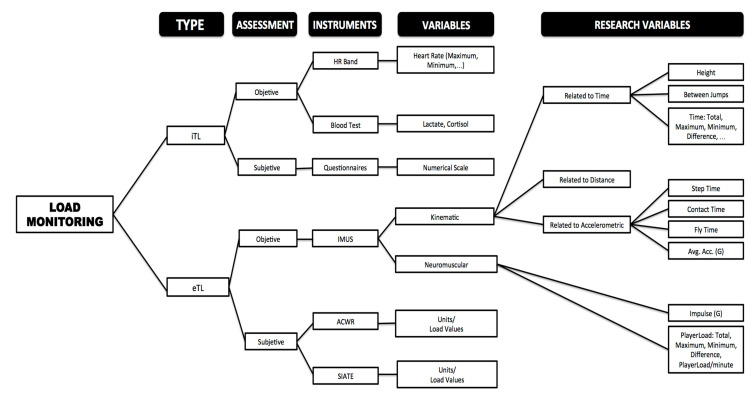
Graphic representation of the variables analyzed according to the type of requirements.

For this research, the age of the players (U14, U16 and U18) and sex (male and female) were defined as independent variables. For the assessment of the lower body strength of the athletes, the following variables were analyzed divided into three groups according to the type of requirements: (i) objective external load kinematic variables related to time or distance, (ii) objective external load neuromuscular variables and (iii) objective external load kinematic variables related to accelerometry [32]. Part of the selected variables has been defined and used in different investigations that share some of the objectives of this investigation [29,33,34]. See Figure 1.

(i) Objective external load kinematic variables related to time or distance. They analyze the external load that the player supports during the execution time and their locomotion.

(i.1) Height: the number of centimeters that the athlete reaches the highest point of the jump.

(i.2) Time: For the Abalakov test and the multi-jump test, the time was measured in milliseconds from the moment of the takeoff to the landing of the jump. In the sprint RSA test, the time it took to perform the 14 m sprint was recorded in seconds. In addition, the following time-related variables were calculated:

(i.2.i) Total time (*Time Total*): the time that the athlete took to perform the five 14-m sprints (expressed in seconds).

(i.2.ii) Average time (*Time Avg*): the average time that the athlete took to perform a sprint, obtained from the average value of the five sprints (expressed in seconds).

(i.2.iii) Maximum time (*Time Max*): the slowest sprint that the athlete performed and the one that took the longest time to travel the 14 m distance (expressed in seconds).

(i.2.iv) Minimum time (*Time Min*): the fastest sprint that the athlete performed and in which the least time was used to cover the 14-m distance (expressed in seconds).

(i.2.v) Time difference (*Time Dif*): the variable that calculates the difference between the fastest and the slowest execution (expressed in seconds).

(i.3) Between jumps: the value of the time that passed between jumps in the multi-jump test expressed in milliseconds. This is the time in which the athlete is in contact with the floor, applying force to carry out another takeoff. It is only calculated in the multi-jump test.

(ii) Objective external load neuromuscular variables. they record the external load that the player supports regarding the gravitational force. Two variables are recorded:

(ii.1) Impulse (*G*): maximum force generated in the action prior to the takeoff of the jump that allows the player to reach the highest possible height, measured through force G.

(ii.2) Player load (*PL*): vector sum of the four accelerometer data points in the three axes of movement (vertical, anteroposterior and lateral). It is represented in arbitrary units (a.u.) and is calculated in the current moment; Xn, Yn and Zn are the values of BodyX, BodyY and BodyZ in the current moment; and Xn–1, Yn–1 and Zn–1 are the values of BodyX, BodyY and BodyZ in the previous moment. Then, the value of player load is calculated and multiplied by 0.01 as a scale factor [35]. The player load variable was relativized per minute for data equality. Moreover, its calculation took into account the following variants related to the player load:

(ii.2.i) Maximum player load (*PL Max*): the sprint in which the athlete supports the highest player load over the 14-m distance.

(ii.2.ii) Minimum player load (*PL Min*): the sprint in which the athlete supports the lowest player load over the 14-m distance.

(ii.2.iii) player load difference (*PL Dif*): the variable that calculates the difference between the sprints that represent the highest and lowest player load.

(iii) Objective external load kinematic variables related to accelerometry: they analyze the external load that the player supports during the execution time, taking accelerometry into account.

(iii.1) Step time: the time that the athlete takes to perform a step in the RSA test run. It is composed of the phase of contact with the floor and the flight phase (expressed in milliseconds).

(iii.2) Contact time: the time that the athlete is in contact with the floor and the foot is supported in the run (expressed in milliseconds).

(iii.3) Flight time: the time that the athlete is in the flight phase of the run (expressed in milliseconds).

(iii.4) Average acceleration (*G*) (*Acc Avg*): the average value of the accelerations performed by the athlete during the sprint.

### 2.4. Performance Test

Three validated tests were carried out to evaluate the explosive strength and tolerance to lower body fatigue that are part of the SBAFIT test battery [3]: the Abalakov test, the multi-jump test and the repeat sprint ability test (RSA test) (5 × 14 m). These tests were selected because they have been designed for the sport of basketball in particular or because they can specifically assess the skills proposed. Therefore, the order was as follows: first Abalakov test, second multi-jump test, and third repeat sprint ability test (RSA test).

○ Abalakov test (ABK) [36]: it consists of a test of maximum lower body strength in which the athlete performs a counter-movement jump with the help of the arms. Each athlete makes three attempts separated by recovery time so that they are not affected by fatigue [3]. Out of the three attempts, the highest jump is selected for analysis. The test was evaluated with the following variables: (i) time; (ii) height; (iii) impulse (G).
○ Multi-jump test (MJ) [3]: it consists of a test that assesses the tolerance to fatigue of the lower body. To do this, the player starts on a box with a height of 50 cm. The player jumps down from the box and makes five maximum jumps in a row using the arm swing. The variables analyzed in this test were time, height, imposed (g), and between jumps (from jump 2 to 5) of each jump. The test was evaluated with the following variables: (i) time; (ii) height; (iii) impulse (G); (iv) between jumps.
○ Sprint test RSA (5 × 14 m) [37]: it consists of a test that assesses sprint speed and tolerance to repeated maximum efforts with incomplete rest. In this test, the athlete performs five sprints in which they must cover 14 m (from the baseline of the basketball court to the midfield line) in the shortest possible time and trying to record the smallest possible difference between repetitions. At the end of each sprint, the player has an active recovery of 30 s. The test was evaluated with the following variables: (i) time total; (ii) time average; (iii) time maximum; (iv) time minimum; (v) time differences; (vi) player load, (vii) player load/minute; (viii) player load maximum; (ix) player load minimum; (x) player load differences; (xi) step time; (xii) contact time; (xiii) flight time; (xiv) average acceleration (G).

### 2.5. Equipment

To record the kinematic objective external load variables related to distance and neuromuscular objective external load variables, each player was equipped with an inertial device model WIMU PRO^TM^ (RealTrack Systems, Almería, Spain), which was fitted using an anatomically adapted harness to every player in the interscapular region. In addition, ChronoJump photoelectric cells (Bosco System, Barcelona, Spain) were used for the kinematic variables related to time. After recording, the data from the inertial devices were analyzed using SPRO^TM^ software (RealTrack Systems, Almería, Spain). The WIMU PRO^TM^ device has been used in this research due to its high validity and precision in the quantification of the analyzed variables [38].

### 2.6. Procedure

First, the clubs and the coaches were contacted to inform them about the project to be carried out. Then, the free moments which each team had in the days prior to participating in the national championship were selected (each category has a different date). The choice of that moment of absence from the competition was selected so that the athletes were in the best physical condition possible. In addition, participants were advised not to engage in any strenuous activity that could alter the results in the 72 h prior to conducting the tests. After the data collection from each team, a dossier was made for the coach with the information from the tests in order to provide greater knowledge about the physical condition of the athletes. To do the tests, the protocol described in the SBAFIT test battery [3] was carried out. Finally, all the study participants carried out a familiarization session with the material to be used in the measurement and were given information on the tests in order to have prior contact with the protocol and equipment so that would not affect the final result of the assessments.

### 2.7. Statistical Analysis

First, performance profiles were made based on age and sex. Next, a descriptive analysis of the quantitative variables (M ± SD) was carried out. Then, criteria assumption tests were performed to determine whether the data were distributed normally [39]. In this case, the results obtained presented a normal distribution, so parametric tests were carried out to contrast the hypothesis. Then, to make the profiles, the values shown were represented through Z-Scores. The purpose of the Z-Scores is to standardize a value so that it represents the number of standard deviations the value is above the mean [26,40]. To identify the significant differences between ages and sex, a MANOVA test with two fixed factors (age and sex) was performed with a post hoc Bonferroni test [41]. Furthermore, the effect size was calculated for MANOVA using partial eta squared (η2) as low effect (0.01–0.06), moderate effect (0.06–0.14) and high effect (>0.14) [42]. SPSS 24.0 software (SPSS Inc., Chicago, IL, USA) was used for the statistical analysis. The significance was established at *p* < 0.05 [39].

## 3. Results

Table 1 shows the descriptive results grouped by age and sex revealing differences. In men players, as the player grows, the results improve (in the RSA test, the time decreases and in the jumping tests, it increases). In women players, unlike men players, the worst results in the RSA test are found in U18 players. In the jumping tests, women players showed a similar evolution to that obtained by men players, but the values were lower.

**Figure 2:**
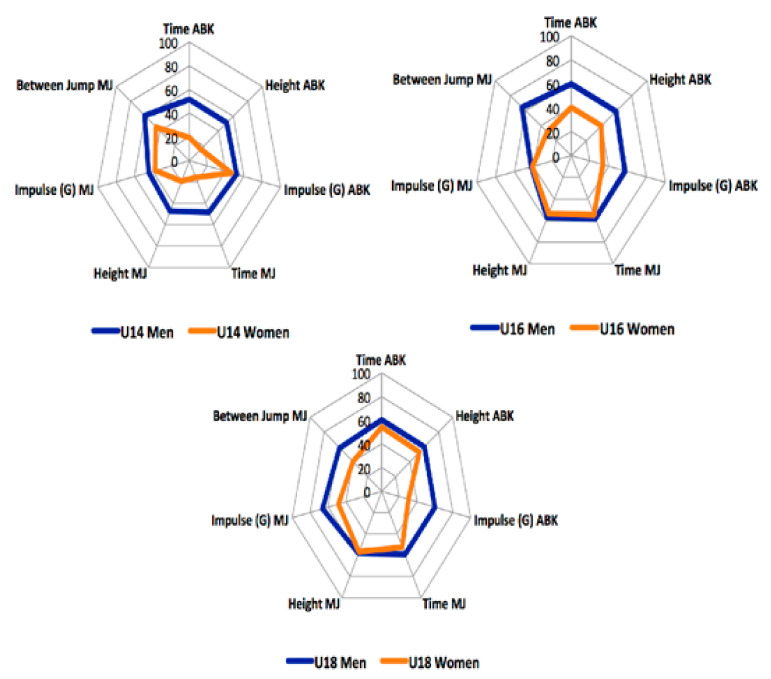
Profiles of performance of the Abalakov (ABK) test and multi-jump (MJ) according to sex and age.

Figure 2 shows the normalized results obtained through the Z-Score grouped according to age and sex in the Abalakov test and the multi-jump test. It was observed that, both in the men and women, as the age of the players increased, the circumference of the profile was greater. This means that as the Z-Score visually shows the represented value that the standard deviation is above the mean. In this case, the larger the graph, the better results obtained by the athletes. Regarding the differences between sexes, men players obtained better results at all ages than women players in the variables analyzed.

**Figure 3:**
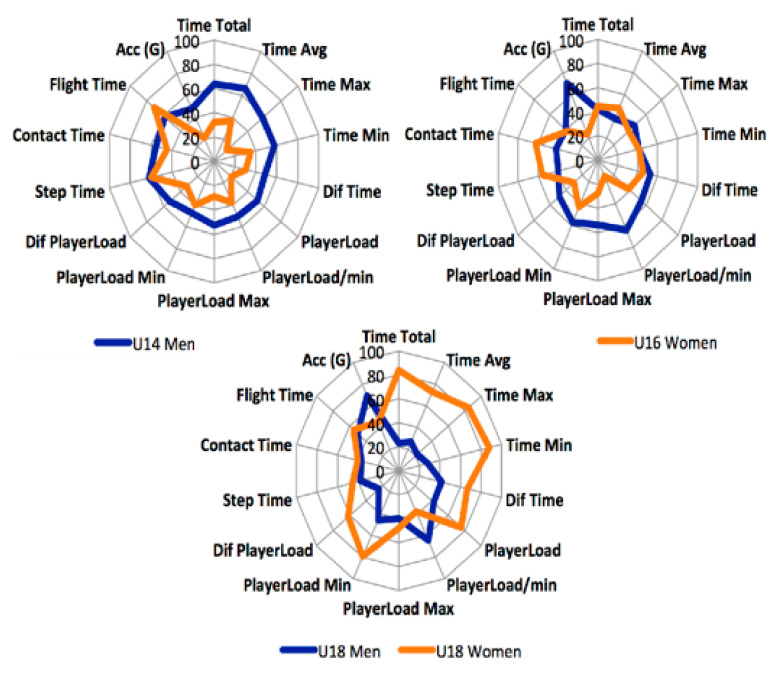
Profiles of performance of RSA test according to sex and age.

Figure 3 shows the normalized RSA test results grouped by age and sex. As in Figure 1, the results are represented through the Z-score. The results show a positive evolution regarding age; the older the age, the better the results. In the RSA test, the best results represent a smaller circumference since they are due in part to variables related to time and the shorter the sprint duration, the faster the player. Regarding gender, men players showed better results than women players of all ages.

Table 2 shows the results of the differences according to age and sex. Regarding age, significant differences were found in most of the variables analyzed. Furthermore, the differences among age groups are revealed by the Bonferroni Post Hoc. In relation to the effect size calculated through partial eta squared, most of the variables are smaller than moderate in size. However, in variables with a very large size, one of the ages is U18. These results affirm that the U18 players are the ones with the greatest differences from the rest of the players. Regarding sex, significant differences are observed in most variables except those belonging to the multi-jump test. The effect size obtained in most of the variables is moderate or low, although some variables of the RSA test stand out with very high values. These variables that present higher values confirm the difference between sexes in the speed test. Based on the results, a larger effect size was found in the variables based on age than on sex.

## 4. Discussion

The objectives of this study were to describe the physical–physiological demands that players faced in different physical fitness tests related to maximum lower body strength and speed to analyze the differences in the selected variables. The main findings of the research show significant differences related to the improvements shown with increasing age, and according to sex, where men players obtain better results in tests than women players. Regarding the relationship of variables, the results reaffirm those existing in the literature showing a relationship between the different variables of speed and lower body strength tests that assess physical fitness [10,13,43]. This research brings new knowledge to the field of training since most research on the assessment of physical fitness is carried out in senior [44] or national teams [45]. There are differences between the results of amateur teams with regard to those obtained in this research by players in formative categories, forcing the coaches or physical trainers to make different adaptations according to the results that affect several principles of training [46]. In this research, the selected sample was made up of athletes from different training categories. In addition, another novel aspect is the inclusion of a reactive strength test in the lower body. For these reasons, the comprehensive assessment of physical fitness in training categories is so important in the development of performance at an early age. These results show a specific profile for each age and sex.

### 4.1. Speed Test (RSA Test)

The results obtained in the speed test (5 × 14 m) showed significant differences based on age and sex. Regarding the kinematic variables related to the sprint execution time, it was observed that it varies depending on the age. Older players covered the distance in less time since their intensity is greater. Coinciding with these findings [21], different distinguished aspects of physical fitness and evolutionary development. Along these lines, the development of anaerobic capacity is related to the physical development of the athlete [47]. This evolutionary process of the athlete influences the development of strength in a limiting way due to its relationship with puberty that causes changes in the size and cross-sectional area of the muscle, affecting muscle contraction and, therefore, the application of strength [48]. These findings confirm that the physical and physiological differences among players must be taken into account to individualize the training process since their responses will be different in the face of the same stimulus. Regarding the variables analyzed according to sex, the obtained results showed significant differences in most variables, with differences between sexes in sprint speed and acceleration capacity, the cause being that strength production is determined by the athlete’s sex. Furthermore, biologically, men players have a greater amount of type II fibers and recruit them to a greater extent. These differences confirm that the production of strength is influenced by the sex of the athlete, and it is common for men players to have a greater number of type II fibers. This morphological difference is a determining factor in the production of strength and power [17,49]. In line with the aforementioned statements and the importance of speed in performance in basketball, ref. [50] confirmed that linear speed is an important parameter in the development of performance in women players, not being so decisive in men players. These differences in physical abilities related to sports performance mean that sports have different characteristics depending on the sex of the athletes.

The relativized neuromuscular load per minute that players endure varies depending on the age. Like the previous variables, due to puberty, older players present higher strength values, and therefore their performance in these tests is higher [48]. These differences are only reflected between sexes at a maximum load; that is, men generate more load than women due to their genetic differences. One of the most important factors in the difference between sexes is linked to body size, in which women have a lower center of mass (smaller body size and smaller limb size) than men, implying lower load strength in the midfoot [51] and greater hip adduction angles [10]. Therefore, the gravitational forces that they support are different. These gravitational forces can be affected not only by sex or the angles of the hips but also by maturational development as puberty causes an increase in weight in the athlete (regardless of sex), and therefore, it affects the load supported.

Finally, in the kinematic variables related to accelerometry, the results obtained showed significant differences in all the variables analyzed according to age. The differences revealed the development and evolution of the cycle of running steps as the athlete develops. In addition, older players also tend to have larger limbs that affect the final sprint result [21]. Regarding the cycle of steps of athletes, the differences among ages are directly related to the development of strength [37]. This variation in strength affects the final result since, in order to apply an equal or greater strength, they use less time and are more efficient, achieving a higher speed of movement. As for the differences between sexes, these variables are directly related to the production of maximum strength and power to obtain greater acceleration. Furthermore, ref. [52] stated that the players of both sexes, when training at maximum performance, show innate differences due to the elastic properties of the muscle. These differences have a direct impact on strength production and power transfer. The results obtained show that men athletes have a different evolution in sprinting ability than women athletes. These differences in evolution affect the planning of the training sessions and the specific work on this ability, which must be individualized based on sex and age.

### 4.2. Lower Body Strength Test (Maximum Strength and Reactive Strength)

In the lower body strength tests performed (Abalakov and multi-jump test), the results obtained show significant differences in both tests depending on age, except for the variable between jumps of the multi-jump test, while significant differences based on the sex of the participants only occurred in the Abalakov test.

Regarding the results of the Abalakov test, they showed differences in all the variables analyzed depending on age. These results coincide with those found in the literature, which confirm the importance of maturational development in the production of maximum strength [53]. As in the RSA test, the evolutionary development and sex of the athlete are of great importance in terms of maximal strength value [54]. The differences between sexes are more evident since the contractile capacity of the muscle to generate maximum strength is higher than to perform a power exercise [55].

In the multi-jump test, significant differences among game categories are found in all the analyzed variables due to the development in puberty of strength production factors [28] except for the variable between jumps, which does not present significant differences among ages. These results may be due to the fact that this variable is related not only to the ability to generate the greatest possible strength in the shortest time but also to the fact that coordination affects execution. On the other hand, no significant differences were found in this test, according to sex. This finding may be because the energy production of a multi-jump test (short application time) requires specific training that the analyzed players do not perform. Ref. [56] stated in their research that sex differences are markedly reduced when subjects are not trained. This statement coincides with the findings that mention the need to perform specific tasks in training to work on lower body reactive strength.

The research has the following limitations. Regarding the sample, the participants were players in a formative period who had a high level of physical fitness to be able to compete in the national championships of each age and sex. Their results may be similar to those of players of the same ages, although their competitive level may condition the result. Data are offered closer to the reality of the sport of the formative period teams. Moreover, this research that analyses the ability to generate strength in the lower body and power in movement speed did not take into account the maturational age of the players, the years of experience or the athlete’s fat-free weight. Only the chronological age was taken into account.

## 5. Conclusions

Specific profiles of physical fitness in young players were identified related to the maximum strength of the lower body and their speed of movement. The results of this research provide new knowledge about the evolution of these abilities according to age and sex. The most important findings show that there is an improvement in skills linked to the athlete’s maturational development. Regarding the results based on sex, men players obtain better values than women players (regardless of age). These differences are because the skills analyzed in this research are related to strength, which, in turn, is influenced by maturational development and sex. The results of these variables provide the field of training with knowledge to individualize the training process based on age or sex to improve the athlete’s physical performance. The reactive strength of young basketball players is not conditioned by sex. The evaluators can select the variables to be used in the evaluation of the physical fitness of the basketball players according to the material resources available (inertial devices, photoelectric cells, etc.). On this occasion, the use of the latest generation of inertial devices facilitated the collection of data, as well as the quantity and quality of the information obtained.

The practical applications of this research are as follows: (i) Older players should work on strength in specific sessions for improvement, while younger players should prioritize strength application exercises. (ii) Work on improving strength levels in specific sessions is not recommended for players who have not undergone the changes of puberty. Regarding the ratio of time and intensity of work and recovery time, older and men players must train these abilities with lower ratios (less rest time), while younger or women players should use higher ratios at work on these abilities (longer rest time per unit of work). (iii) The study brings practical knowledge to the field of training in a developing evolutionary population. Therefore, to improve jump height in this test, not only is lower body strength training necessary but also, specific and coordinative training must be carried out to improve this action.

By jointly analyzing all the contributions identified in the different tests, our results have shown that there is a greater influence on the physical performance of athletes from their maturational state than because of their sex. This evidence was revealed by Ramos when he identified the influence of maturational development on the performance of young basketball players. The differences found between players of different sexes in the same age range are not as relevant as those found with regard to age, as there are age ranges where the difference in performance is not so evident (before puberty).

## Figures and Tables

**Table 1:** Descriptive results of the analyzed variables grouped by age and sex.

		U14 Men		U14 Women		U16 Men		U16 Women		U18 Men		U18 Women	
Performance Test	Variable	Mean	SD	Mean	SD	Mean	SD	Mean	SD	Mean	SD	Mean	SD
Repeat sprint ability (RSA)	Time total	14.13	1.08	13.83	0.64	13.58	1.81	14.12	0.95	13.27	0.83	14.98	0.73
	Time avg.	2.82	0.22	2.77	0.13	2.60	0.36	2.82	0.19	2.65	0.17	2.99	0.15
	Time max.	3.04	0.25	2.91	0.13	3.05	0.44	3.03	0.20	2.86	0.20	3.17	0.28
	Time min.	2.60	0.29	2.60	0.12	2.56	0.28	2.61	0.18	2.48	0.18	3.70	0.16
	Time dif.	0.44	0.27	0.31	0.14	0.49	0.25	0.42	0.16	0.38	0.18	0.47	0.19
	PL	1.42	1.02	0.95	0.25	1.70	1.72	0.97	0.61	1.22	0.26	1.67	0.27
	PL/min.	4.84	1.08	4.15	1.11	5.73	1.27	4.14	0.39	5.65	1.14	4.41	0.69
	PL max.	0.50	0.98	0.21	0.15	0.66	1.67	0.21	0.15	0.28	0.06	0.37	0.05
	PL min.	0.21	0.06	0.18	0.08	0.23	0.05	0.18	0.11	0.20	0.07	0.30	0.06
	PL dif.	0.29	1.01	0.34	0.16	0.43	0.66	0.32	0.16	0.81	0.58	0.69	0.36
	Step time	283.91	11.35	282.59	10.92	266.39	21.13	290.77	46.61	266.92	16.28	272.47	18.91
	Contact time	204.34	14.50	197.00	14.13	194.95	13.82	223.11	35.41	190.37	14.86	193.60	14.69
	Flight time	79.56	7.58	84.86	7.86	71.43	10.21	67.66	15.33	76.40	12.82	78.86	10.20
	Acc (G)	2.38	0.38	1.97	0.31	2.93	0.50	1.89	0.40	2.97	0.56	2.35	0.27
Abalakov	Time (ms)	512.95	51.46	454.50	35.40	540.54	67.07	472.95	72.70	551.49	86.03	518.17	47.48
	Height (cm)	32.64	6.36	25.47	3.97	36.37	8.84	28.04	8.65	38.18	11.34	33.19	5.90
	Impulse (G)	2.73	1.63	2.31	1.30	2.95	1.06	2.08	0.61	3.26	1.35	2.09	0.49
Multi-jump	Time	459.80	47.77	404.88	34.15	484.00	50.19	475.05	46.04	488.00	53.55	469.38	49.85
	Height	26.41	5.79	20.67	4.27	29.39	5.95	28.13	5.38	29.92	6.62	29.00	5.31
	Impulse (G)	3.24	1.05	3.19	2.04	3.13	0.96	3.04	0.71	4.06	0.67	3.46	0.78
	Between jump	535.50	97.43	464.72	81.42	572.71	122.00	370.12	122.74	518.00	81.21	441.52	83.79

Time max.: maximum time in the sprint (slowest sprint); Time min.: minimum time in the sprint (fastest sprint); Time dif.: difference between the fastest and the slowest sprint; PL max.: maximum player load in a sprint; PL min.: minimum player load in a sprint; PL dif.: difference between the maximum and minimum player load; Step time: total time of contact time and flight time; Contact time: contact time on the floor; Flight time: time in the air; Acc avg step (G): average acceleration during the sprint (measured in G-force); Time jump: duration of the jump; Height: maximum height reached in the jump; Impulse (G): impulse performed (measured in G-force); Between jumps: time between jump and jump (contact time on the floor).

**Table 2:** Results of the differences according to age and sex of the analyzed variables.

		Age			Sex		Age*Sex	
Performance Test	Variable	Sig.	Eta	Post Hoc	Sig.	Eta	Sig.	Eta
RSA Test	Time total	<0.001 *	0.762	U14-U18	<0.001 *	0.488	<0.001 *	0.452
	Time avg	<0.001 *	0.715	U14-U16	<0.001 *	0.565	<0.001 *	0.681
	Time max	<0.001 *	0.659	U14-U16; U16-U18	<0.001 *	0.009	<0.001 *	0.787
	Time min	<0.001 *	0.688	U14-U16; U14-U18	<0.001 *	0.446	<0.001 *	0.792
	Time dif	0.002 *	0.089		0.292	0.002	<0.001 *	0.175
	Player load	0.391	0.218		0.446	0.012	0.216	0.012
	Player load/min	0.023 *	0.117	U14-U16; U16-U18	<0.001 *	0.001	0.387	0.151
	Player load max	0.48 *	0.011		0.203	0.013	0.188	0.025
	Player load min	<0.001 *	0.221	U14-U16; U16-U18	0.761	0.044	<0.001 *	0.326
	Player load dif	0.465	0.012		0.193	0.048	0.375	0.015
	Step time	0.014 *	0.063	U14-U18	0.016 *	0.004	0.029	0.053
	Contact time	<0.001 *	0.127	U16-U18	0.011 *	0.421	<0.001 *	0.131
	Flight time	<0.001 *	0.184	U14-U16; U16-U18	0.481	0.128	0.152	0.029
	Acc avg step (G)	<0.001 *	0.200	U14-U16; U14-U18	<0.001 *	0.132	0.002 *	0.089
ABK Test	Time	<0.001 *	0.132	U14-U18	<0.001 *	0.001	0.894	0.002
	Height	<0.001 *	0.138	U14-U18	<0.001 *	0.069	0.985	0.000
	Impulse (G)	0.019 *	0.059		0.693	0.060	0.001 *	0.099
MJ Test	Time	<0.001 *	0.231	U14-U16; U14-U18	0.002 *	0.002	0.045 *	0.047
	Height	<0.001 *	0.219	U14-U16; U14-U18	0.005 *	0.259	0.104	0.034
	Impulse (G)	0.018 *	0.060	U14-U18	0.607	0.156	0.003 *	0.084
	Between jumps	0.184	0.026		<0.001 *	0.219	0.002 *	0.090

*: *p* < 0.05; ABK Test: Abalakov test; MJ test: multi-jump test; Time Total: total time of 5 sprints; Time avg: average sprint time; Time max: maximum sprint time; Time min: minimum sprint time; Time dif: difference between the fastest and the slowest sprint; player load/min: player load per minute; PL max: maximum player load in a sprint; PL min: minimum player load in a sprint; PL dif: difference between the maximum and minimum player load; Step time: total time of contact time and flight time; Contact time: contact time on the floor; Flight time: time in the air; Acc Avg step (G): average acceleration during the sprint (measured in G-force); Time jump: duration of the jump; Height: maximum height reached in the jump; impulse (G): impulse performed (measured in G-force); Between jumps: time between jump and jump (contact time on the floor).

## Data Availability

The data presented in this study are available on request from the corresponding author. The data are not publicly available due to the participants in this research are minors and that Organic Law 3/2018, of 5 December, on the Protection of Personal Data and Gaurantee of Digital Rights of the Government of Spain requires this information is in custody.
